# Institutionalized elderly: vulnerabilities and strategies to cope with Covid-19 in Brazil

**DOI:** 10.17533/udea.iee.v39n1e07

**Published:** 2021-03-05

**Authors:** Pricila Oliveira de Araújo, Maria Yaná Guimarães Silva Freitas, Evanilda Souza de Santana Carvalho, Thaís Moreira Peixoto, Maria Lúcia Silva Servo, Laiane da Silva Santana, Juliana Macêdo dos Santos Silva, Jenny Caroline Vieira Moura

**Affiliations:** 1 Nurse, Master. State University of Feira de Santana, Feira de Santana, Bahia - Brasil. Email: poaraujos@gmail.com Universidade Estadual de Feira de Santana State University of Feira de Santana Feira de SantanaBahia Brazil poaraujos@gmail.com; 2 Nurse, Doctoral. State University of Feira de Santana, Feira de Santana, Bahia - Brasil. Email: yana@uefs.br Universidade Estadual de Feira de Santana State University of Feira de Santana Feira de SantanaBahia Brazil yana@uefs.br; 3 Nurse, Post-Doctoral. State University of Feira de Santana, Feira de Santana, Bahia - Brasil. Email: evasscarvalho@yahoo.com.br Universidade Estadual de Feira de Santana State University of Feira de Santana Feira de SantanaBahia Brazil evasscarvalho@yahoo.com.br; 4 Nurse, Master. State University of Feira de Santana, Feira de Santana, Bahia - Brasil. Email: thaismorep@hotmail.com Universidade Estadual de Feira de Santana State University of Feira de Santana Feira de SantanaBahia Brazil thaismorep@hotmail.com; 5 Nurse, Doctoral. State University of Feira de Santana, Feira de Santana, Bahia - Brasil. Email: mlsservo@uefs.br Universidade Estadual de Feira de Santana State University of Feira de Santana Feira de SantanaBahia Brazil mlsservo@uefs.br; 6 Nurse, Master student. Federal University of Bahia, Salvador, Bahia - Brasil. Email: laianesantana00@gmail.com Universidade Federal da Bahia Federal University of Bahia SalvadorBahia Brazil laianesantana00@gmail.com; 7 Nursing student. State University of Feira de Santana, Feira de Santana, Bahia - Brasil. Email: jully495.jm@gmail.com Universidade Estadual de Feira de Santana State University of Feira de Santana Feira de SantanaBahia Brazil jully495.jm@gmail.com; 8 Nursing student State University of Feira de Santana, Feira de Santana, Bahia - Brasil. Email: jennycvmoura@gmail.com Universidade Estadual de Feira de Santana State University of Feira de Santana Feira de SantanaBahia Brazil jennycvmoura@gmail.com

**Keywords:** coronavirus infections, institutionalization, elderly, infecciones por coronavirus, institucionalización, anciano, infecções por coronavírus, institucionalização, idoso

## Abstract

This article presents a systematized reflection and discussion around two guiding axes: the first discusses aging and vulnerabilities to biological, physical, cognitive, social and affective losses that require specific attention, as well as vulnerabilities to COVID-19 to which institutionalized elderly people are exposed; the second, we reflect on the adoption of restrictive and protective measures to prevent the spread of the virus, aiming to keep the elder health and mitigate the effects of the pandemic. The conclusion is that the pandemic has increased the many vulnerabilities to which institutionalized older people were already exposed, adding vulnerability to a new disease, such as COVID-19, due to its high lethality and comorbidity, aggravated by precariousness of long-term Brazilian institutions due to the negligence of public authorities, civil society, the management of the institution and the families of the patients. The post-pandemic scenario will require collective efforts to protect and ensure the survival of the elderly living in those residences.

## Resumen

Este artículo presenta una reflexión y discusión sistematizada en torno a dos ejes orientadores: en el primero, se habla sobre el envejecimiento y las vulnerabilidades a las pérdidas biológicas, físicas, cognitivas, sociales y afectivas que requieren atención específica, y, así mismo, sobre las vulnerabilidades a COVID-19 a las que están expuestos los ancianos institucionalizados; en el segundo eje, se reflexiona sobre la adopción de medidas restrictivas y de protección para prevenir la propagación del virus con el objetivo de mantener la salud del anciano y mitigar los efectos de la pandemia. La conclusión es que esta ha incrementado las numerosas vulnerabilidades a las que ya estaban expuestas las personas mayores institucionalizadas, agregando, además, la vulnerabilidad a una enfermedad nueva como es el COVID-19, debido a la alta letalidad y comorbilidad que representa, la cual, es agravada por la precariedad de las instituciones brasileñas de larga estancia debido a la negligencia de las autoridades públicas, la sociedad civil, la gerencia de la institución y las familias de los pacientes. El escenario post-pandemia requerirá esfuerzos colectivos para proteger y garantizar la supervivencia de los ancianos que viven en estas residencias.

## Resumo

Este artigo apresenta uma reflexão sistematizada e discussão em torno de dois eixos orientadores: o primeiro discute o envelhecimento e vulnerabilidades a perdas biológicas, físicas, cognitivas, sociais e afetivas que requerem atenção específica, bem como vulnerabilidades à COVID-19 a que os idosos institucionalizados são expostos; e na segunda, refletimos sobre a adoção de medidas restritivas e protetivas para prevenir a propagação do vírus, com o objetivo de manter a saúde dos idosos e mitigar os efeitos da pandemia. A conclusão é que a pandemia aumentou as muitas vulnerabilidades às quais os idosos institucionalizados já estavam expostos, agregando vulnerabilidade a uma nova doença, como a COVID-19, devido à alta letalidade e comorbidade que representa, agravada pela precariedade das instituições brasileiras de longo prazo devido à negligência do poder público, da sociedade civil, da gestão da instituição e das famílias dos pacientes. O cenário pós-pandemia exigirá esforços coletivos para proteger e garantir a sobrevivência dos idosos que vivem nessas residências.

## Introduction

Since the new coronavirus, named SARS-CoV-2, appeared and spread on all continents, the world has started a race to know and control the disease that causes COVID-19 and that has caused widespread fear in people and crises in several segments, especially sanitary. This disease has a wide clinical spectrum ranging from asymptomatic infections to severe conditions, as it is rapidly spread and potentially fatal, it represents the most important worldwide public health problem in the last 100 years.(1) The risk of dying from COVID-19 increases with age and the presence of comorbidities, since most deaths occur in the elderly, especially those with chronic diseases.

Until September 5, 2020, at the end of Epidemiological Week number 36, 4,123,000 cases and 126,203 deaths in the general population were confirmed in Brazil, of which 75% of the victims were elderly, thus showing that the risk of death from the disease increases with advancing age.(2^,^^3^) Elderly people affected by COVID-19, who have geriatric syndromes and/or other diseases, may suffer a weakening process that leads to physical-cognitive dependence. Consequently, there is a need for these elderly people to be cared for by third parties, who may be partners, children or another family member and, at this moment, difficulties may arise due to the lack of financial or environmental conditions to subsidize care. Thus, the Long Term Care Facilities (LTCF) appear, as an alternative to guarantee basic care to this population.(4)

LTCF in the Brazilian context are governmental or non-governmental institutions, of a residential nature, intended for the collective home of elderly people with or without family support, under conditions of freedom, dignity and citizenship.(5) The main advantages are to protect elderly people who suffer abuse or other violence at home, supporting them in a safe place, with basic health care and providing conditions to keep them alive, with food, housing and a hygienic environment.(6) As disadvantage, institutionalization generates social exclusion for the elderly and creates barriers for the establishment of strengthened human relationships, which promotes consequences for the organism as a whole, such as the development or enhancement of muscle weakness, cognitive impairment, aggravation of non-communicable disease, excessive sadness that can lead to depression, loss of communication skills, among others(7) Thus, this process of losses and gains affects the elderly in different ways and depends on culture, socioeconomic factors, family and social support networks, as well as the facilities and difficulties that the elderly person faced throughout of life.(6)

In Brazil, a census conducted by the Institute of Applied Economic Research (IPEA) in 2011, indicated the existence of about 90 thousand elderly people living in 3,600 institutions in Brazil, corresponding, at the time, to almost 1% of the country's elderly population, being that the majority of these ILPIs (65%) were philanthropic.(8) Then there are the private ones, which represent 28.2% of the total of institutions.(9) In a national survey conducted between 2016 and 2018, it was identified that approximately 51 thousand elderly people lived in public and philanthropic institutions in the country, 65% of which were semi-dependent or dependent and, therefore, fragile; In 2020, that number seems to be around 78 thousand elderly people.(2)

Most elderly people living in these places have basic conditions for survival, access to health services/resources and a place to live until finitude arrives. Thus, given the peculiarities surrounding the institutionalization process, the repercussions of this process on the physical and mental health of the elderly, the pandemic of the new coronavirus and the high lethality of COVID-19 in this population, it is important to reflect on the vulnerability to which institutionalized elderly are subjected, as well as to discuss the coping strategies of COVID-19 that will become necessary during and after the pandemic. The objective is to reflect on the various vulnerabilities to which institutionalized elderly people are exposed, as well as on strategies for coping with COVID-19.

## Institutionalized elderly people and the vulnerabilities enhanced by the COVID-19 pandemic

The aging process occurs in an individual and heterogeneous way, however with the approach of old age, the tendency is for the human organism to become more vulnerable to aggressions from the internal and external environment, occurring biological, physical, cognitive, social, affective losses. The symbolic, social and cultural dimensions that one has about this stage of life and that are linked to the chosen paths and the determinants of aging will influence the ability to have an autonomous and independent old age, that is, with a maintained functionality.(10) In addition, previous illnesses, comorbidities, lifestyle, access to health services also influence the elderly person's ability to manage their own lives.

Many elderly people need to resort to LTCF for survival; it is known, however, that rich, remedied, or poor elderly people who are in a state of chronic pathologies or dementia, whose care offer has become impossible at home, also seek these institutions.(11) To have the assistance of workers who work at these institutions can be considered a good thing or not, depending on the perspective, individual characteristics,(6) life history and resilience of the elderly. The vast majority of institutionalized people have chronic illnesses that make them fragile and unable to perform self-care and personal hygiene practices. In addition, they share the same sources of air, food, water, caregivers and medical care among themselves and, in addition to the turnover of caregivers and workers in the same environment, become more susceptible to infections.(12) This intersection intrinsic and extrinsic factors predispose them to contamination by SARS-CoV-2 and other infectious agents that cause respiratory, dermatological and other diseases.

It is noteworthy that many elderly people are unable to live independently, which demands the need for continuous and daily supplementary care by caregivers (which increases the chance of contracting COVID-19) and the mobilization of resources, often inadequate due to the lack of financial and professional qualifications.(13) With this, elderly people enjoy a minimum health care offer, in which, in reality in public or philanthropic institutions in Brazil, health professionals perform punctual and prioritize the most serious cases of illness, as a consequence of the biomedical model that still persists as the most adopted in Brazil. Nursing care is performed with a focus on attendance the basic human needs of Wanda Horta, such as nourishing, hydrating and sanitizing.(14)

In turn, the atypical clinical course of COVID-19 as the possibility of absence of fever, and which, combined with the challenge or impossibility of conducting an interview, in the case of elderly people with neurocognitive disorder, and the presence of comorbidities, can delay diagnosis and treatment of the disease. Consequently, it impairs health status surveillance, facilitates viral dissemination within the institution and provides clinical complications.(15) In addition, the signs and symptoms manifested by elderly people can be understood by workers as “things of age”, the which generates trivialization of complaints or discredit due to episodes of delirium, cognitive disorders and others.

On the other hand, the circulation of visitors and workers in the LTCF favors the transmission of pathogens from the community to the institution.(16) In the current period of the pandemic, in which the elderly are assisted by caregivers and workers who have wide circulation whether in other institutions or in the community in general, the risk becomes more evident, which motivated managers to carry out strict screening of COVID-19 in these spaces and to temporarily prohibit visits to the LTCF. This decision led to the interruption of group activities that are mostly carried out by religious visitors, groups of students or even volunteers who carried out different activities at the LTCF and who, with the pandemic, suffered an abrupt rupture. These measures, despite being the most effective to guarantee social isolation and, therefore, reduce the chances of becoming ill, generate in elderly people more and more feelings of loneliness, abandonment and discouragement, which can worsen the clinical stage of pre-existing diseases, increasing the risk of depression, weight loss and disturbing behavior.(6) In addition, family members are important agents to monitor the quality of care provided at LTCFs. With their limitation and staff absenteeism due to contagion and the need for quarantine, the quality of care, already considered low in many institutions, probably decreased further in this pandemic period.(12)

As most LTCF have few workers and there is a need to adopt quarantine, there may be a deficit in the workforce for the provision of care and for the supervision that the team does to the semi-dependent and independent elderly when performing self-care. Therefore, one of the greatest challenges to be overcome in these pandemic times is to guarantee the quality of the services provided, since their discontinuity, such as food, medication, hygiene, dressings, can compromise the survival of institutionalized elderly people. A worker or elderly person infected with SARS-CoV-2 in a long-term institution also means a high chance of transmission to others, as caregivers work very close to the elderly. These factors contribute to the outbreak of COVID-19 in these locations, being associated with high rates of morbidity and mortality, due to the clinical characteristics of people and the routine of institutions.(16)

But there are other aspects that increase the vulnerability of the elderly to infection by COVID-19, such as the LTCF infrastructure and the scarcity of resources. The first factor is related to the fact that LTCF are not designed or equipped to deal with deaths from COVID-19, as they do not have adequate space for the isolation of dead bodies. This occurs, not through negligence, but because these places are not hospitals, but homes for the elderly(12) and, as such, solutions need to be thought out for possible needs. In addition, many LTCF has an excess of residents, which makes it difficult to establish the necessary distance. The scarcity of resources is related to the adequacy and sufficiency of Personal Protective Equipment (PPE) for workers at the LTCF, insufficient rapid tests even for elderly people recently admitted for diagnostic screening, which should be done regularly,(17) as well as hygiene materials of adequate quality and quantity and few resources for infection control and prevention.

Given the above, it is clear that the challenge of reducing the vulnerabilities of institutionalized elderly people and protecting them from COVID-19 is not small. This is partly because, in Brazil, LTCF are neglected by the government, which invests very little in these units, leaving philanthropy to protect the elderly people who live there: abandoned elderly people; victims of negligence or ill-treatment; or with weakened family ties and who depend exclusively on the care provided by workers at the LTCF.

The State has a duty to implement laws and public policies for all citizens regardless of age, color and ethnicity and which must be followed and executed successfully. Sometimes, people's fundamental rights are not being respected, such as: dignity, access to decent housing, food and security. Elderly people, as they are more vulnerable to aggressions from the external and internal environment, suffer more from the denial of these rights and resort to family members and they, in turn, seek the LTCF as a viable option for each type of situation. The reflections that arise during the development of this work lead us to realize the importance of the State to guarantee the social protection to which every Brazilian citizen is entitled, whether him is inside or outside an LTCF. Currently, the protection of institutionalized elderly people in COVID-19 is evident and urgent, since an outbreak of the disease in an LTCF means a considerable increase in infected people and even an abbreviation of life span, therefore requiring of special attention regarding investment in coping strategies, which will be the topic discussed below.

## COVID-19 coping strategies in Long-Term Care Facilities for the Elderly

Since the beginning of the pandemic, the World Health Organization(18) has developed protocols for the prevention of COVID-19 in institutions for the elderly. Among the measures, include the training of caregivers; guidance on preventive practices; promoting hand hygiene and respiratory etiquette; ensuring availability of supplies available to prevent COVID-19; early recognition of symptomatic cases; social isolation; and adequate specialized assistance, when necessary. The United States' Center for Disease Control and Prevention has also suggested that LTCF develop a comprehensive response plan, providing for restricted visits, removing unnecessary health care and canceling group activities, including community meals.(15^,^^19^)

A study carried out in Taiwan, Republic of China, summarized the infection control measures to be used in the context of care for the elderly: 1) Designate a director responsible for training the team, as well as for the communication, planning and monitoring of prevention activities and combating COVID-19; 2) keep updated vaccination cards, routine exams and identification of flu-like symptoms, both in the elderly and in workers; 3) discourage workers from working in more than one institution to prevent the spread of disease; 4) provide sufficient PPE; 5) cleaning and disinfecting environments as a routine practice followed by two steps, the first with ordinary detergent and the second with bleach for hospital use; 6) develop a traffic control package at the LTCF in order to minimize the risk of COVID-19.(15)

Of the measures listed above, that of discouraging workers from working in more than one institution can be considered a measure of unfeasible applicability, since there is a precarious salary and lack of appreciation for occupations of this nature. At the Catalan Center Geriàtric, in Lleida, an important way of preventing COVID-19 in the institutionalized elderly population was that 24 workers agreed to isolate themselves with their 89 residents.(20) Obviously, this is a different reality from that found in Brazilian institutions, in which workers must work in two or even three different locations to supplement family income. This measure to discourage multiple bonds requires a wide appreciation for working hours and wages compatible for the subsistence of workers. In addition, the spread of the virus promotes illness and implies absenteeism in teams that require a resizing of personnel in these scenarios. Consequently, when it is not possible to replace the sick worker, there is an overload of the others and a deficit in the quality of care. Another recommendation to avoid community meals requires a reorganization of sufficient and adequate physical space for a distance of 1.5 m among the elderly or the reorganization of food supply schedules with alteration of the routine in the LTCF. Traffic control aims to limit the movement of people who have been exposed to contacts with foreigners, sick people and others. In the reality of community transmission in which we live in Brazil, such control refers to anyone who is not a resident of the LTCF and requires adequate materials to carry out a detailed screening of the people who circulate in the institution, as well as adequate physical space for the organization of the transit of people.

For this traffic control to take place, it is necessary to carry out a detailed anamnesis of those who enter the facilities and categorize them as follows: belonging to the transition zones, which are regions destined for people who come from other hospitals; and clean areas where there is no transit of health workers. Those who work in the clean zone should not attend cafeterias and meetings; the place must be disinfected before and after the work shift; keep social distance and, in case of need to be in a group, keep at least two meters away and always use masks. In case of the need for a meeting with the residents, in addition to maintaining distance and use of PPE, time should be used at an average of 30 minutes.(15) It is important to reflect that in addition to the adequate material conditions for the implementation of these measures, there is also a need for education of all those involved in the process (workers and elderly people) so that they understand the importance of it and strictly comply with the determinations. In the case of elderly people with cognitive impairment, supervision is required in case of non-compliance with the regulations. That is, there is no point in organizing the COVID-19 coping protocol within the LTCF if workers and elderly people do not understand the importance of it. There needs to be a permanent education process so that people have the clarity that the circulation of the virus will continue for a long time in Brazil and, consequently, the coping measures as well.

Thus, there are measures that will take a while to be implemented and perhaps they should be permanently incorporated, such as: installing hand disinfection sites in various points of the LTCF; in the case of specialized assistance, one should avoid going out with the elderly as much as possible; avoid daytime programming for non-resident elderly people; if there is a need to leave the LTCF, hand hygiene measures must be used, masks should be worn by all occupants of the vehicle, prevent the driver from passing through more than one health institution, take care of the driver's health and history of contacts. Any resident who leaves the facility must be quarantined for two weeks after returning to the facility, which requires space and additional staff, and it is not always possible in the Brazilian reality that LTCF have only one driver on the workforce ( as) and small physical spaces that hinder isolation.(15)

If elderly people need to buy drugs or need up-to-date medical prescriptions, they must be provided by staff, so that they do not have to leave the institution. These measures are the gold standard and it is known that most LTCF cannot meet these recommendations. But one must try to get closer and closer to the ideal for the prevention of COVID-19 in LTCF with feasible measures, reaffirming respect for human beings in situations of fragility.

When COVID-19 occurs in elderly people at the LTCF, it is necessary to offer psychological support to workers who are in direct care for sick people; strengthening communication between workers and family members, being essential to minimize the deleterious effects of family leave during the period of stay at the LTCF; The implementation of a palliative approach to the necessary cases must be taken into account, following ethical principles to guarantee quality of life, comfort and dignity for the resident.(21) Regarding the elderly in a situation of palliative care and their families, one must offer the possibility to decide on how to proceed regarding the care plan in situations of finitude. The individual decision should be discussed with close people who visit the elderly and needs to be documented and always accessible, for example, in emergency situations. If a resident decides not to go to the hospital, palliative care should be planned in the institution's environment.(22) In view of the difficulties of the LTCF to maintain continuous and quality work in non-pandemic times, this shortage is exacerbated in pandemic times. So, it is necessary to use recreational activities that provide entertainment and distraction, as well as support with financial help from civil society and local companies, in order to meet basic demands that contribute to coping with COVID-19. Local action aimed at maintaining health for the institutionalized population should be advocated, with a view to mitigating the effects of the pandemic.(23) To mitigate the deleterious effects of social isolation, it is also necessary to integrate technological advances, such as videoconferences. , chat apps, among others that promote the socialization of the elderly with family members and visitors, and others that improve health perception and promote physical activity.(24)

## Conclusions

Reflection on elderly people living in long-term institutions, whether philanthropic or public, allows us to state that they experience, from a very early age, the harmful effects of social isolation, the absence of affectivity and family contact, of idleness and sedentary lifestyle that end up for generating fear, deep sadness, depression, decompensation and worsening of heart disease, muscular and skeletal problems, neurological, among others. In other words, institutionalized elderly people were always more vulnerable to the factors listed above, but with the pandemic, the vulnerability to illness by COVID-19 became more intense and worrying. This is due to the high lethality of this disease at older ages, the presence of comorbidities and the precarious situation that many Brazilian LTCF undergo, due to the negligence of the public authorities, civil society, the management of these units and families. The threat of coronavirus contamination and the impossibility of implementing effective measures to prevent and cope with COVID-19 within institutions make control options quite limited and, consequently, penalize elderly people who need to become even more socially isolated for protect yourself from contamination. Furthermore, we need to reflect on the post-pandemic scenario and recognize that measures to combat COVID-19 will need to be implemented in Brazil for a long time, which will require efforts by the government, maintainers, family members and caregivers to protect and guarantee survival. elderly people who depend on LTCF.

## References

[1] Medeiros EAS (2020). Desafios para o enfrentamento da pandemia Covid-19 em hospitais universitários. Rev. Paul. Pediatr..

[2] Sociedade Brasileira de Geriatria e Gerontologia (2020). Brasil ultrapassa 100 mil óbitos por Covid-19 Idosos são 75% das vítimas.

[3] Ministério da Saúde (BR) (2020). Secretaria de Vigilância em Saúde. Boletim epidemiológico especial. Doença pelo Coronavírus COVID-19. Brasilia (DF).

[4] Cavalcanti AD (2013). Envelhecimento e institucionalização: uma revisão bibliográfica à luz da promoção da saúde. Rev. Kairos Gerontol..

[5] Agência Nacional de Vigilância Sanitária (2005). Resolução - RDC nº 283.

[6] Oliveira JM, Rozendo CA (2014). Instituição de longa permanência para idosos: um lugar de cuidado para quem não tem opção?. Rev. Bras. Enferm..

[7] Lini EV, Portella MR, Doring M (2016). Fatores associados à institucionalização de idosos: estudo caso-controle. Rev. Bras. Geriatr. Gerontol..

[8] Brasil. Secretaria de Assuntos Estratégicos da Presidência da República (2011). Comunicados do IPEA. Série Eixos de Desenvolvimento Brasileiro nº93.

[9] Alcântara AO, Camarano AA, Giacomin KC (2016). Política nacional do idoso: velhas e novas questões.

[10] Pereira DS, Nogueira JAD, Silva CAB (2015). Qualidade de vida e situação de saúde de idosos: um estudo de base populacional no Sertão Central do Ceará. Rev. Bras. Geriatr. Gerontol..

[11] Duarte LMN (2014). O processo de institucionalização dos idosos e as territorialidades: espaço como lugar?. Estudios Interdisciplinares sobre o Envelhecimento.

[12] Gardner W, States D, Bagley N (2020). The Coronavirus and the Risks to the Elderly in Long-Term Care. J. Aging. Soc. Policy.

[13] Utsumi M, Makimoto K, Quroshi N, Ashida N (2010). Types of infectious outbreaks and their impact in elderly care facilities: a review of the literature. Age Ageing.

[14] Horta WA (1974). Enfermagem: teoria, conceitos, princípios e processo. Rev. Esc. Enf..

[15] Yen MY, Schwartz J, King CC, Lee CM, Hsueh PR (2020). Recommendations for protecting against and mitigating the COVID-19 pandemic in longterm care facilities. J. Microbiol. Immunolog. Infect..

[16] Lai CC, Wang JH, Ko WC, Yen MY, Lu MC, Lee CM (2020). COVID-19 in long-term care facilities: An upcoming threat that cannot be ignored. J. Microbiol. Immunolog. Infect..

[17] Iacobucci G (2020). Covid-19: Lack of PPE in care homes is risking spread of virus, leaders warn. BMJ.

[18] World Health Organization (2020). Infection prevention and control guidance for long-term care facilities in the context of COVID-19 interim guidance.

[19] Centers for Disease Control and Prevention (2020). Preparing for COVID-19 in Nursing Home.

[20] Oliver D, Oliver David (2020). Let's not forget care homes when covid-19 is over. BMJ.

[21] Etard JF, Vanhems P, Atlani-Duault L, Ecochard R (2020). Potential lethal outbreak of coronavirus disease (COVID-19) among the elderly in retirement homes and long-term facilities. Euro Surveill.

[22] Kunz R, Minder M (2020). COVID-19 pandemic: palliative care for elderly and frail patients at home and in residential and nursing homes. Swiss Med. Wkly.

[23] Xiao Y, Torok ME (2020). Taking the right measures to control COVID-19. Lancet Infect. Dis..

[24] Eghtesadi M (2020). Breaking Social Isolation Amidst COVID‐19: A Viewpoint on Improving Access to Technology in Long‐Term Care Facilities. J. Am. Geriatr. Soc..

